# Inhibition of* In Vivo* Growth of* Plasmodium berghei* by* Launaea taraxacifolia* and* Amaranthus viridis* in Mice

**DOI:** 10.1155/2016/9248024

**Published:** 2016-12-05

**Authors:** Adewale Adetutu, Olubukola S. Olorunnisola, Abiodun O. Owoade, Peter Adegbola

**Affiliations:** Department of Biochemistry, Ladoke Akintola University of Technology, PMB 4000, Ogbomoso, Nigeria

## Abstract

*Launaea taraxacifolia* and* Amaranthus viridis* used by people of Western Africa in the treatment of malaria and related symptoms were assessed for their antiplasmodial value against the chloroquine sensitive strain of* Plasmodium berghei*. Crude extracts (200 mg/kg) and chloroquine (5 mg/kg) were administered to different groups of Swiss mice. The percentage of parasitemia, survival time, and haematological parameters were determined. Both extracts significantly (*p* < 0.05) inhibited parasitemia and improved survival time in infected mice. The crude extracts prevented loss of some haematological parameters.* A*.* viridis* had a distinct effect on the packed cell volume. The extract was able to protect the liver from some of the damage. This study however showed that the methanolic extracts of* A. viridis* and* L. taraxacifolia* possess antiplasmodial activity. The results of this study can be used as a basis for further phytochemical investigations in the search for new and locally affordable antimalarial agents.

## 1. Introduction

Malaria remains one of the major causes of death worldwide with about 600 million people at risk of infection [1, 2]. The majority of the mortality from malaria is caused by infection with* Plasmodium falciparum*, which have been reported to pose the greatest risk to nonimmune individuals and children [3]. Malaria is widespread in the Sub-Saharan Africa with more than 90% of the population at risk [4, 5].

The use of the conventional drugs in the treatment of malaria has been exasperated by the resistance of the* P*.* falciparum* to most of the recognized antimalaria drugs [6–8]. Therefore, medicinal plants have been major complementary natural therapeutic remedies used for treatment of malaria infection in many developing countries [9, 10]. The use of plant materials for treatment might be imperative and beneficial as key important and active ingredients of antimalarial drugs (artemisinin and quinine derivatives) are known to have their source from herbs [11].

*L. taraxacifolia* and* A. viridis* are widely consumed as food supplements in many parts of Western Africa. These vegetables are rich in anti-infective phytochemicals, micronutrients, vitamins, and antioxidant components [12, 13]. These vegetables are also known for their protective role in oxidative stress related disorders [14] and as dietary antioxidants in augmenting cellular defences [12, 15]. The investigation of these vegetables in the treatment of malaria is scarce. Therefore, this study assessed the effectiveness of* L. taraxacifolia* and* A. viridis* extracts in the reduction and prevention of blood plasmodial levels and hepatocellular damage in mice infected with* P. berghei*.

## 2. Materials and Method 

### 2.1. Plant Collection and Extract Preparation

Fresh leaves of* L. taraxacifolia* and* A. viridis* were obtained from the Ladoke Akintola University Agricultural Farm, Ogbomoso, Nigeria. The plants were identified and authenticated by Prof. Ogunkunle J. A. of the Department of Pure and Applied Biology LAUTECH, Ogbomoso, with voucher numbers LHO 231 and LHO 233 for* L. taraxacifolia* and* A. viridis*, respectively. The plant materials were air dried at room temperature and then powdered. 130 g of extracts was weighed and soaked in 450 mL methanol for 72 hrs with constant shaking. The mixture was filtered separately using Whatman paper of 150 mm diameter. The residues were discarded and the filtrate was collected and concentrated using a rotary evaporator [16].

### 2.2. Treatment and Infection of Mice

Twenty-five male Swiss mice (25–35 g) aged 6–8 weeks were purchased from the Animal House, Department of Pharmacy, Obafemi Awolowo University, Ile-Ife, Nigeria. These animals were acclimatized for a period of 2 weeks in groups of five in cages with wooden shaves for bedding materials. They were fed with grower mash and water. Permission and approval for animal experiment were certified by the Animal Ethnics Committee, Faculty of Basic Medical Sciences, Ladoke Akintola University of Technology, Ogbomoso. Rodent parasite,* P. berghei*, was used in this study and the parasites were maintained through serial passage in mice. Cardiac blood sample from the donor mouse with percentage of parasitemia of 59.385% was used. The blood sample was diluted with normal saline such that 0.2 mL of 1 × 10^7^ *P. berghei* infected erythrocytes was inoculated intraperitoneally into each experimental mouse [17].

### 2.3. Determination of Percentage of Parasitemia

The percentage of parasitemia was determined using the methods of Kalra et al. [18]. From the tail of infected mice, thin smears were prepared on slides. The slides were allowed to dry and then fixed with methanol. After fixing, the slides were allowed to dry and then stained with 10% Giemsa in methanol for 30 mins. After 30 mins, the slides were rinsed with water and then allowed to dry. To estimate the percentage of red blood cells infected with malaria parasites, the slides were carefully observed under microscope using ×100 objective with immersion oil in 10 different fields on each slide. The % parasitemia was calculated using the formula

(1) $$\begin{array}{c} \%\;parasitemia=\frac{no\;\;of\;\;parasitized\;\;RBC}{total\;\;no\;\;of\;\;RBC}\times 100. \end{array}$$

Also the % inhibition of the parasite was calculated for each group by the formula

(2) $$\begin{array}{c} \%\;\;Inhibition=\frac{mean\;\;\%\;\;parasitemia\;\;of\;\;untreated\;\;group-mean\;\;\%\;\;parasitemia\;\;of\;\;treated\;\;group}{mean\;\;\%\;\;parasitemia\;\;of\;\;untreated\;\;group}\times 100. \end{array}$$

The animals throughout the period of the experiment were under careful watch. Observations such as change in fur, appearance, agitation, colour, odour and colour of urine, faeces, weight, and other physical observations were noted.

### 2.4. Experimental Design and Treatment of Mice

Methods of Peter et al. [19] and Kalra et al. [18] for antiplasmodial assay against* P. berghei* infection in mice with some modifications were employed. Twenty-five infected mice were randomly divided into five groups (two experimental and three control groups), each having five mice (Table 1). The animals in each group except for the control and the negative control groups were orally pretreated with 200 mg/kg/body weight of the extracts for two weeks. This was done in order to check if the extracts possess protective ability against malaria. Each mouse was inoculated intraperitoneally with* Plasmodium berghei* ANKA strain parasites. The inoculum was prepared from a donor mouse with rising parasitemia of 30–45%. After 9 days of infection, animals begin to receive treatment (200 mg/kg b wt.) for two weeks with constant check of the percentage of parasitemia at four-day interval. 0.2 mL of chloroquine (5 mg/kg b wt.) was used as positive control and 0.9% DMSO in distilled water as negative control. The extract dosage was prepared by first dissolving in 0.9% dimethyl sulfoxide (DMSO) in distilled water. All the extracts and the drugs were given orally by using a standard intragastric tube. Also, alternatively, another group was infected on day 0 and began treatment on the 10th day of infection with the starting percentage of parasitemia noted being also grouped. The mice stopped receiving treatment after 16 days of treatment. This was assessed to verify if the extract could cure or suppress malaria. For all parasitemia determination, blood samples were collected from tail snip of each mouse and thin smears prepared and stained with 10% Giemsa solution. Five uniform fields of each stained slide (for each mouse) were examined under microscope with an oil immersion objective of 100X magnification power and average percent of parasitemia was determined. Then, group average percent of parasitemia was calculated and used to determine percent or curative suppression with respect to the negative controls.

### 2.5. Haematological Parameter Analysis

Twenty-four hours after the last dose on the 16th day of infection, the animals were sacrificed by cervical dislocation and the blood samples were collected by heart puncture. The blood samples for haematological parameters (red blood cell (RBC) count, white blood cell (WBC) count, platelet count, packed cell volume (PCV), and haemoglobin (HGB)) were collected into EDTA bottles and analysed using an automated machine (Automated CBC Analyser: Sysmex KX-21).

### 2.6. Histological Study

The liver was obtained from each mouse, washed, and then fixed in 10% formal saline. The fixed tissues were then embedded in paraffin, sectioned (5 *μ*m) with a rotary microtome, and stained with haematoxylin and eosin (H&E). The liver sections were evaluated histologically with a camera attached to a light microscope (Nikon E400). The extent of* P. berghei*-induced liver damage was evaluated based on pathologic lesions in liver sections stained with H&E method.

### 2.7. Statistical Analysis

Percent of suppression of parasite growth of the treated and control groups was compared using one-way ANOVA and two-tailed Student's *t*-test (Graph Pad Prism 4.0, Graph Pad Software, San Diego, USA), with *p* < 0.05 being considered significant.

## 3. Results

### 3.1. Estimation of Percentage of Parasitemia

**Figure 1:**
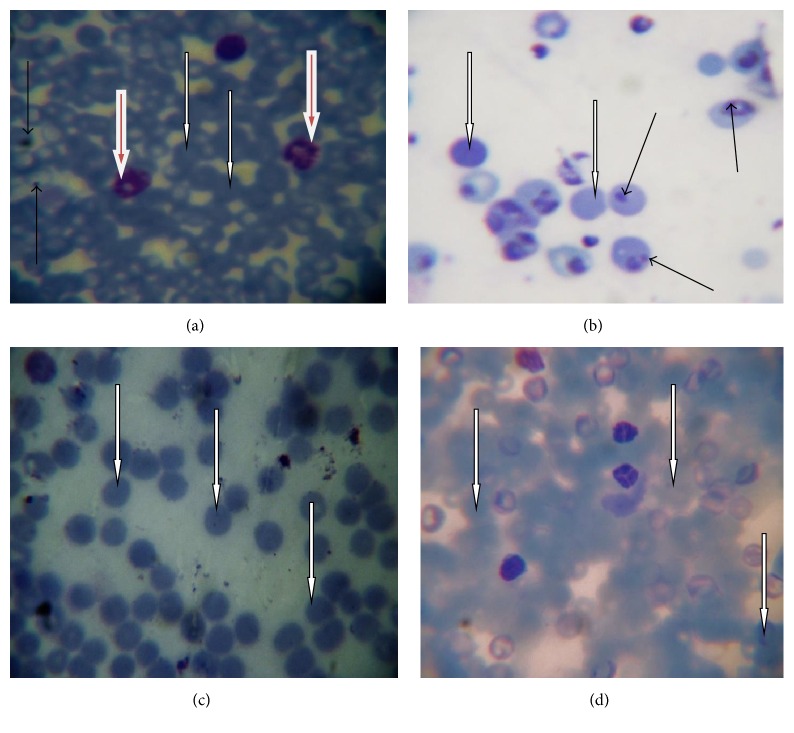
Selected representative of photomicrograph of thin blood smear stained with Giemsa. The thin black arrows point to the parasitized RBC; white arrow indicates normal RBC; the brown arrow points to the WBC. (a) Positive control; (b) untreated group; (c) receiving* L*.* taraxacifolia*; (d) receiving* A*.* viridis* original magnification, ×100.

**Figure 2:**
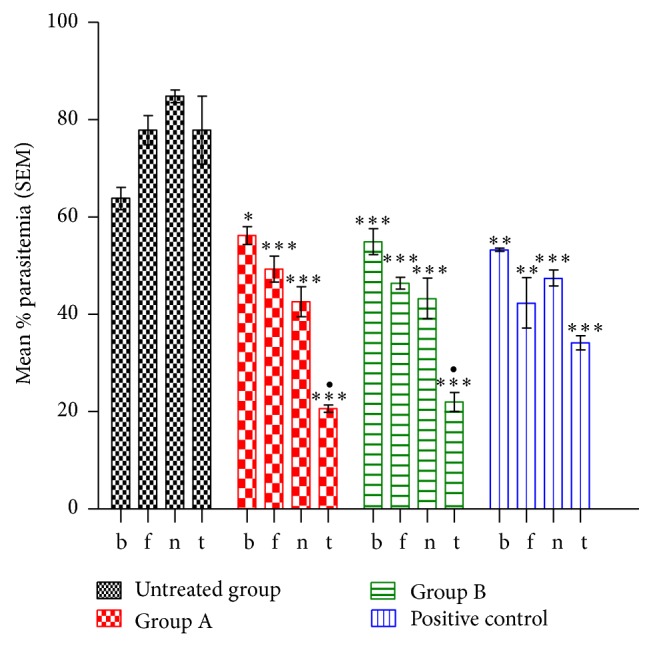
Percentage of parasitemia in* P. berghei* infected mice with decrease in parasitemia in the treated groups. ^*∗∗∗*^Significant difference when compared to the negative control group; ^•^significant difference when compared to the positive control group. Keys: B: before treatment. F: fourth day of treatment. N: eighth day of treatment. T: twelfth day of treatment. ^*∗*^means values are significantly different at *p* < 0.05 when compared with the positive control group. ^*∗∗*^means values are significantly different at *p* < 0.05 when compared with the untreated group.

Figure 1(b) showed numerous parasitized RBC and few normal RBC in mice infected with* P*.* berghei* alone, while the mice infected with* P. berghei* and treated with chloroquine,* L. taraxacifolia,* and* A. viridis* displayed many normal RBC and few parasitized cells (Figures 1(a), 1(c), and 1(d)). In addition, Figure 2 showed the percentage of parasitemia of mice infected with* P. berghei*. However, significant reduction (*p* < 0.05) was observed on days four, eight, and twelve of treatment in the treated groups in comparison with the untreated group. There was no significant difference (*p* < 0.05) on days four and eight of treatment when the positive control group was compared to the* L. taraxacifolia* treated group and* A. viridis* treated group except on day twelve. Overall, Figure 2 presents the percentage of parasitemia in* P. berghei* infected mice with the untreated group exhibiting increase in percentage of parasitemia but a decrease in the treated groups as the treatment proceeds.

#### 3.1.1. Parasite Inhibition and Survival Time

Table 2 showed the assessment of inhibition of* P. berghei* in mice. The percentage of parasite inhibition in all the treated groups increases on days 8 and 12 of treatment (Table 2). However, there is no significant difference (*p* > 0.05) when the* L. taraxacifolia* (Group A) treated group and the* A. viridis *treated group were compared with the positive control group. The groups that received* L. taraxacifolia* (Group A) and* A. viridis *(Group B) and chloroquine after infection with* P. berghei* recorded no mortality after 12 days of treatments. The survival time in* P. berghei* infected mice after 12 days of the experiment showed that the highest mortality rate was recorded in the untreated group.

### 3.2. Haematological Parameter Analysis

Table 3 indicated the result of the haematological parameters of* P. berghei* infected mice after treatments.* L. taraxacifolia* group recorded the highest WBC count of (7.4 ± 6.40), followed by the chloroquine group (6.07 ± 0.18).* A. taraxacifolia* group recorded the highest platelet count while the untreated group exhibited the lowest. The HGB and RBC count in the negative control group was the least while the chloroquine group recorded the highest count in the two parameters followed by the* A. viridis *group. Neither the extracts nor the chloroquine significantly prevented the reduction of PCV as compared to the untreated group.

### 3.3. Histology

**Figure 3:**
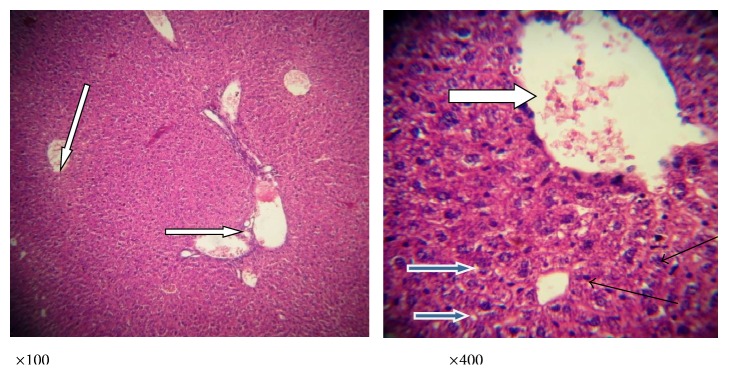
Photomicrograph of a liver section of mice fed with distilled water, no parasite or extracts. The liver was stained with haematoxylin and eosin showing normal liver architecture as seen in ×100 magnification; the sinusoids (slender arrow) are normal without infiltration of inflammatory cells; the central vesicles (white arrow) appear normal and not congested. The hepatocytes (blue arrow) appear normal with normal nuclei and cytoplasmic morphology. There is no haemorrhage and no infiltration of inflammatory cells.

**Figure 4 fig4:**
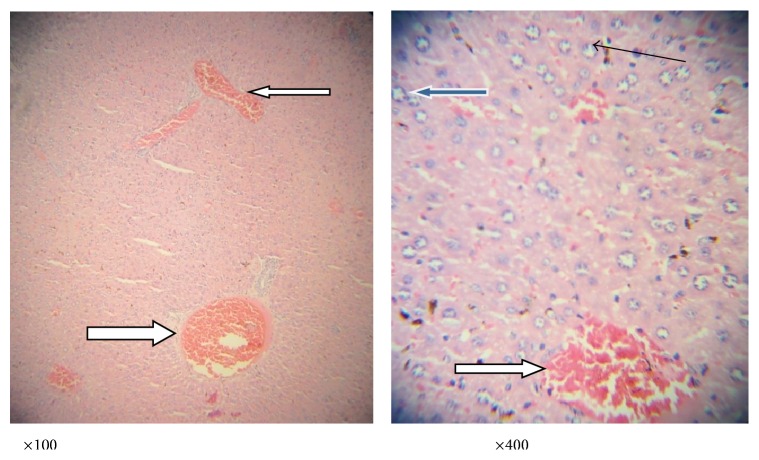
Photomicrograph of a liver section of mice infected with* P*.* berghei* only showing congested central veins and portal vein (white arrow). There is no moderate periportal infiltration by inflammatory cells; there is mild infiltration of sinusoids by inflammatory cells (slender arrow); the hepatocytes morphology shows very few vesicular nuclei (blue arrow).

**Figure 5 fig5:**
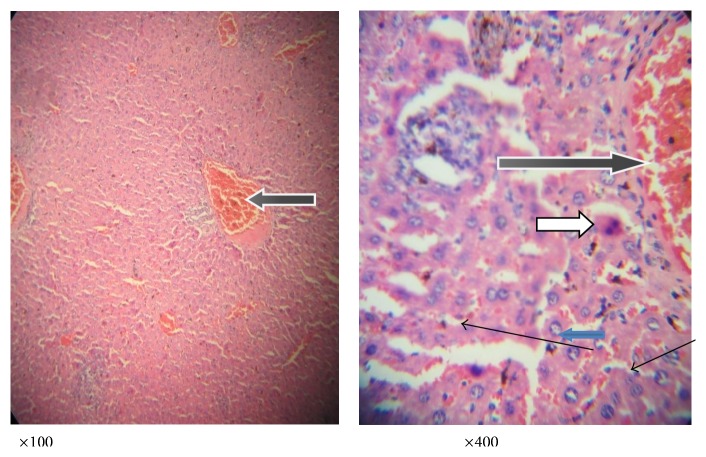
Photomicrograph of a liver section of mice infected with* P*.* berghei* and treated with chloroquine showing mildly dilated sinusoids with inflammatory cells and diffused red cells (slender arrow). The portal vein is congested (black arrow). There is mild periportal infiltration by lymphocytes; most of the hepatocytes morphology appears normal while other few show vesicular nuclei (blue arrow); also seen are binucleated apoptotic hepatocytes (white arrow).

**Figure 6 fig6:**
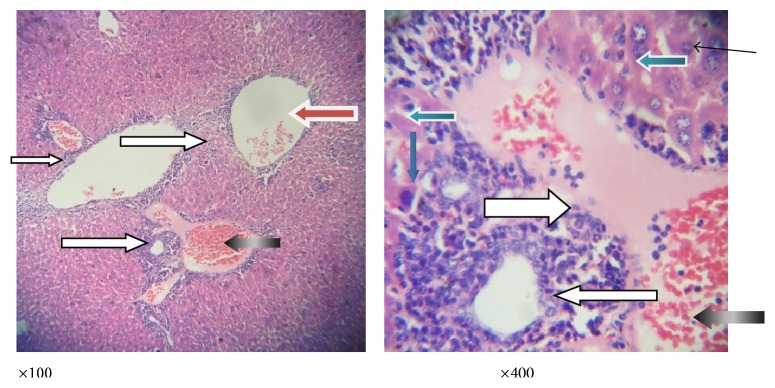
Photomicrograph of a liver section of mice infected with* P. berghei* and treated with* L*.* taraxacifolia* showing moderate perivascular infiltration (white arrow); there is mild infiltration of sinusoids by inflammatory cells (slender arrow). Some hepatocytes appear big and exhibit vesicular nuclei and coarse chromatin (blue arrow).

**Figure 7:**
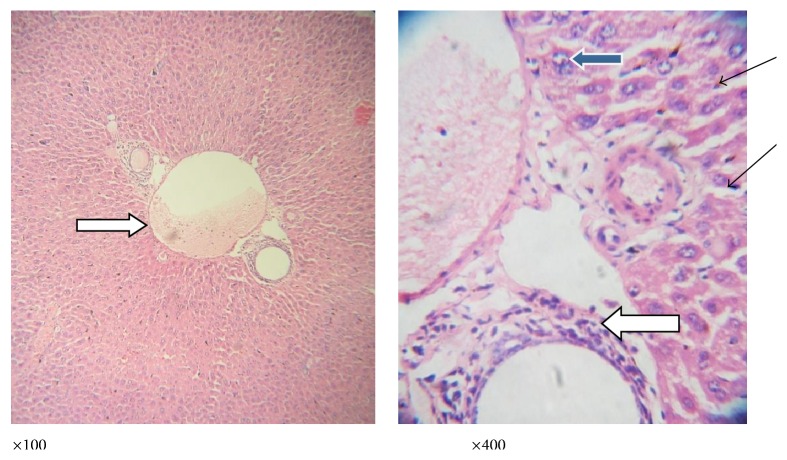
Photomicrograph of a liver section of mice infected with* P*.* berghei* and treated with* A*.* viridis* showing no vascular congestion; there is no infiltration of the portal vein; however, the portal veins are mildly inflamed (white arrow). There are scanty inflammatory cells infiltrating the sinusoids (slender arrow); few hepatocytes exhibit vesicular nuclei (blue arrow); others appear normal.

Figures 3 [Fig fig4] [Fig fig5] [Fig fig6]–7 are the photomicrographs of the lesions induced by* P. berghei* treatment and oral treatments with 200 mg/kg/day of methanolic extract of* L. taraxacifolia*,* A. viridis,* and chloroquine methanolic extract of* L. taraxacifolia*.* P. berghei* infection was marked by hepatic centrilobular vacuolation and vascular congestion indicative of hepatic necrosis (Figure 4) when compared to normal hepatic architecture (Figure 3). However, treatments with 200 mg/kg/day of methanolic extract of* L. taraxacifolia*,* A. viridis,* and chloroquine caused amelioration of the* P. berghei*-induced liver inflammation (Figures 3 [Fig fig4] [Fig fig5] [Fig fig6]–7).

## 4. Discussion

Malaria is one of the most disturbing parasitic diseases arguably affecting the whole developing world, producing serious financial havoc, and impeding the progress of these countries. The situation is dreadful since we have neither a consistent drug against malaria nor a known vaccine that is not yet restrained through drug resistance by the malaria parasite. To overcome the challenge of resistance against the obtainable antimalarial agents, medicinal plants can be a key source of discovering active components for better efficacy. In this context, this research work assessed the curative and the suppressive capability of extracts of* L. taraxacifolia* and* A. viridis* on established malaria infection. In both assays, the evaluation of the percent of inhibition of parasitemia is the most reliable parameter. Additionally, the hepatoprotective activity and alteration effect of the extracts on haematological parameters were assessed after treatment.

The leaves of* L. taraxacifolia* are reported to have hypolipidaemia effect and also the ability to treat water retention disorders [20, 21], respiratory problems, chest congestion, haemorrhoids [22], hepatitis [23], and asthma [24].* In vitro* antioxidant activity of* L. taraxacifolia* has been reported [25]. In Southwestern Nigeria,* A. viridis* is cooked slightly and used in the treatment of haemorrhoids [26]. Furthermore, the plant possesses antiproliferative and antifungal as well as antiviral properties [27]. Anti-inflammatory activity [28], antioxidant activity [29], antimicrobial activity [29], and hepatoprotective activities of* A. viridis* [30] have been reported.

In this study, we described the effects of extracts of* L. taraxacifolia* and* A. viridis* in elimination of malaria parasite in* P. berghei* infected mice.* L. taraxacifolia, A. viridis*, and chloroquine significantly showed antimalaria activity against chloroquine sensitive* P. berghei* infection in mice as evidenced by the percentage of parasite inhibition. The percentage of parasite clearance was very low during the first week of treatment with the methanolic extract of* L*.* taraxacifolia* and* A*.* viridis* but higher during the last week of treatment. It is interesting to observe that the inhibition by the extracts was better than the positive control, an established drug (chloroquine) used for treatment of malaria. These results showed that the vegetables had* in vivo* antimalarial activity and did suppress the multiplication of* P*.* berghei* parasites in mice, an indication that these extracts are a potential source for new antimalarial drugs.

Although the active ingredients in these vegetables for the antimalarial effects are not known or yet to be identified, the presence of alkaloids, glycosides, tannins, and flavonoids [31] has been implicated in antiplasmodial activity and might be as a result of a single additive or synergistic action of these compounds [32]. The alkaloids possess antiplasmodial properties. The most well-known alkaloid for the treatment of malarial is quinine. Alkaloids, phenolic compounds, and terpenoids previously identified in these plant extracts could be responsible for their antimalarial activity. Another possible mechanism for the antimalaria efficacy of the extracts might be related to the immune strengthening property of these phytochemicals. For example, flavonoids have been reported to possess potential immune-modulatory effects [33].

Evaluation of the complete blood count provides enormous information on the haematological status in disease condition [34]. Anaemia is usually assessed by evaluating the packed cell volume (PCV), haemoglobin (HGB), and red blood cell (RBC) count [35] in malaria patients. This study evaluated the changes in haematological parameter in mice treated with the extracts after infection with* P*.* berghei*. The result of this study showed an insignificant decrease in these parameters in the untreated group when compared with the treated and control groups. This is consistent with anaemia seen in malaria. An unusually low HGB concentration is implicative of anaemia [35]. Consequently, it might be that the extract has antianaemic properties. However, the control group has the highest value for this parameter followed by the* A. viridis* treated group and then the* L. taraxacifolia* group. This implies that* A. viridis* according to this study is a better antianaemia extract than* L. taraxacifolia*. The clearance or destruction of infected RBC, the clearance of uninfected RBC, and erythropoietic suppression and dyserythropoiesis have also been implicated in human and in mouse malarial anaemia [36]. Consequently, PCV was measured in this study to assess the efficacy of the extract and chloroquine in preventing haemolysis due to a rising parasitemia level.

Extract of* A. viridis* significantly prevented PCV reduction. Prevention of PCV reduction could probably be associated with the presence of phytochemicals in the extracts, which have strong antihaemolytic effects [37]. WBC as well as other cells are involved in the body's immune system and help to fight disease [35]. Though an increase in WBC has been demonstrated to be linked to severe malaria [36], during acute malaria, WBC counts are generally observed to be low or normal [38]. The results of this study recorded an insignificant difference in the WBC counts of all groups. In addition, the platelet count in* L. taraxacifolia* treated group was insignificantly higher when compared with the other groups. The results of this study showed that the extracts enhanced the normal status of the WBC and platelets.

Several studies have shown that damage to the liver could occur during the erythrocytic stage in the life cycle of the malaria parasite [39]. It is known that several inflammatory stimuli, including the immune response to infectious agents, can lead to liver injury [40–42]. Pathogens like* Propionibacterium acnes* and* P. berghei* are also known to be capable of inducing acute inflammation in the liver [43, 44]. Consequently, this study examined the effects of* P. berghei* infection and treatment with methanolic extract of* L. taraxacifolia* and* A. viridis* on the liver of mice. In this study, the liver histology of* P. berghei* infected mice revealed sinusoid infiltration by inflammatory cells, periportal infiltration by lymphocytes and polymorphonuclear cells, changes in the hepatocytes, portal vein congestion, and central vein congestion, as well as vascular congestion. Overwhelming of immune response has been implicated in severe malaria. The group treated with the extract and chloroquine had a mild infiltration of sinusoids by inflammatory cells, which might be as a result of the enhancement of stimulation of anti- and proinflammatory immune response to the parasite [45, 46].

It can be established from this study that the methanolic extracts of* L. taraxacifolia* and* A. viridis* had significant inhibitory activity against chloroquine sensitive* P. berghei* in mice. Consequently, this study confirms the effectiveness of the leaves of* L*.* taraxacifolia* and* A*.* viridis* as antiplasmodial when used as food supplements. Continuous usage of* L. taraxacifolia* and* A. viridis *may be useful dietary supplements in the prevention and treatment of malaria. Moreover, bioguided fractionation of the vegetables may reveal the active ingredients that might be helpful in treating malaria. To the best of our knowledge, this is the first report on the antimalarial activity of the extracts of* L. taraxacifolia* and* A. viridis*.

## Supplementary Material

Graphical Absract: Inhibition of *in*-*vivo* Growth of *Plasmodiumberghei* by *Launaeateraxacifolia* and *Amaranthusviridis* in Mice.

## Figures and Tables

**Table 1:** Grouping of animals and treatment.

Groups	Treatments
Control	No infection, no treatment
A	Infected with *P*. *berghei* and treated with *Launaea taraxacifolia*
B	Infected with* P. berghei *and* Amaranthus viridis*
C	Infected with *P*. *berghei* and treated with chloroquine (positive control)
D	Untreated infected control (negative control). They were fed with the normal feed and water

**Table 2:** Percentage of parasite inhibitory activity of the methanolic extract of *L. taraxacifolia* and *A. viridis *on *P. berghei*.

Test group	Parasite inhibition (%)			Survival time/days
	4th day of treatment	8th day of treatment	12th day of treatment	
Negative control	0	0	0	7.01 ± 2.01
Group A	36.75	49.76^*∗*^	73.49^*∗*^	19.13 ± 1.95^*∗*^
Group B	40.37	48.97^*∗*^	71.76^*∗*^	20.42 ± 2.01^*∗*^
Positive control	45.60	44.04	56.12	20.54 ± 3.01

Data are expressed as means ± SEM; *n* = 4.

^*∗*^Indicating significance at level of *p* < 0.05 as compared to animal treated with chloroquine.

**Table 3:** Haematological parameters in *P. berghei* infected mice treated with *L. taraxacifolia* and *A. viridis* with the control group.

Parameters	Groups				
	Control	Positive control (chloroquine)	Negative control (no treatment)	Group A (*L. taraxacifolia*)	Group B (*A*. *viridis*)
WBC (×10^9^/L)	6.07 ± 0.18	4.1 ± 1.14	5.20 ± 0.23	7.4 ± 6.40	5.05 ± 1.75
HGB (g/dL)	12.75 ± .0.57	9.25 ± 1.33	8.80 ± 1.32	10.6 ± 0.60	12.5 ± 0.41
RBC (×10^12^/L)	8.42 ± 0.34	6.24 ± 0.79	5.76 ± 0.56	7.3 ± 0.46	8.02 ± 0.71
PLT (×10^3^/*µ*L)	486.67 ± 52.69	304 ± 114.49	408 ± 112	793 ± 355	544 ± 83.24
PCV (%)	43.4 ± 1.86	31.4 ± 3.95	29.20 ± 0.10	35.9 ± 3.70	41.35 ± 2.35

WBC: white blood cells; HGB: haemoglobin; RBC: red blood cells (erythrocyte count).

PLT: platelets count; PCV: packed cell volume.
