# Supplementary material for: Inhibition of In Vivo Growth of Plasmodium berghei by Launaea taraxacifolia and Amaranthus viridis in Mice
Source: Malar Res Treat. 2016 Dec 5;2016:9248024. doi: 10.1155/2016/9248024 (PMC5165229; doi:10.1155/2016/9248024)
Supplement: Supplementary file 1 — Graphical Absract: Inhibition of in-vivo Growth of Plasmodiumberghei by Launaeateraxacifolia and Amaranthusviridis in Mice. [file 9248024.f1.docx]

Inoculation of mouse with *Plasmodium berghei*


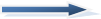


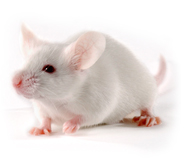

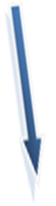


***
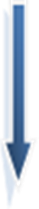
* Extracts of L. *teraxacifolia* or A. *viridis treatment* *
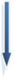
***

**( Feeding of extracts caused suppressive and curtive effects on mice)**


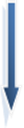

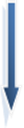


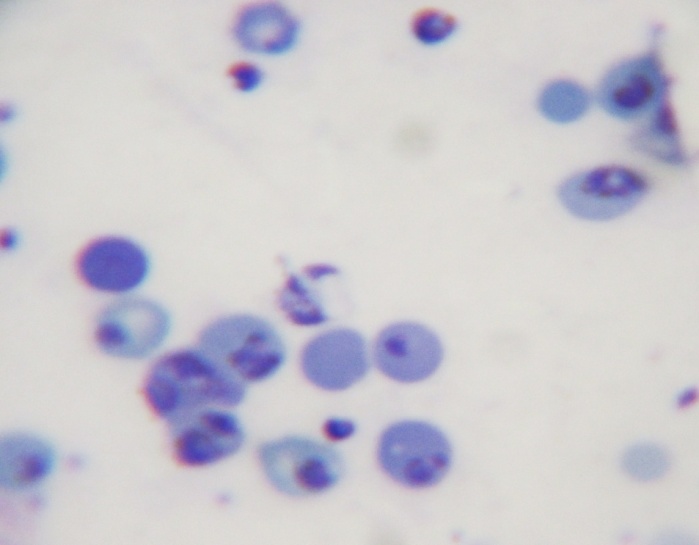

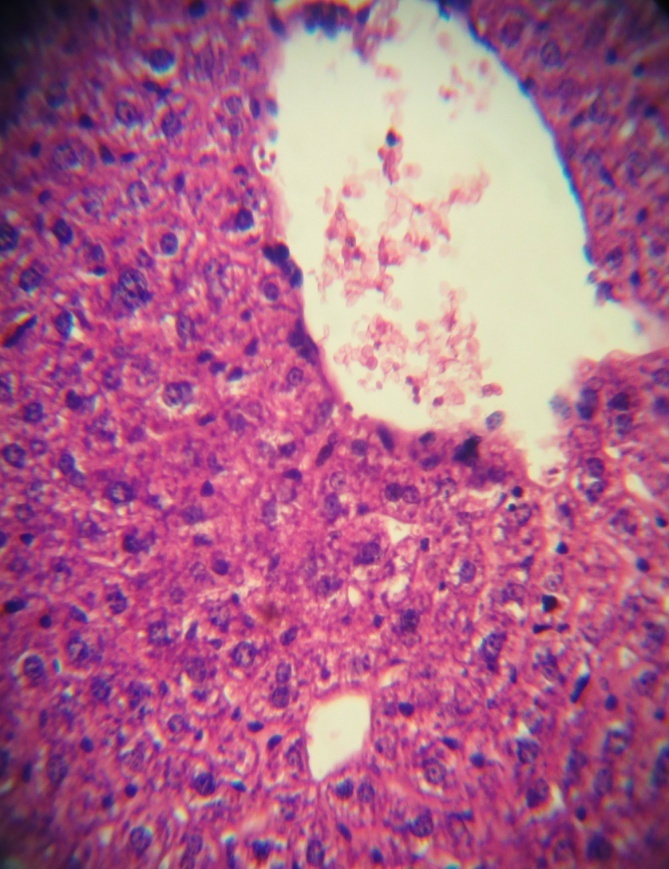


(Paracitised and normal red blood cells) (inflammation and lession on liver tissue)

**Graphical Absract: Inhibition of *in*-*vivo* Growth of *Plasmodium* *berghei* by *Launaea* *teraxacifolia* and *Amaranthus* *viridis* in Mice**
